# Predictors of unintended pregnancy in Kersa, Eastern Ethiopia, 2010

**DOI:** 10.1186/1742-4755-9-1

**Published:** 2012-01-12

**Authors:** Nega Kassa, Yemane Berhane, Alemayehu Worku

**Affiliations:** 1 College of Health Science, Haramaya University, Harar, Ethiopia; 2 Addis Continental Institute of Public Health, Addis Ababa, Ethiopia; 3 School of Public Health, Addis Ababa University, Addis Ababa, Ethiopia

**Keywords:** Pregnancy, Intention, Unintended, Mistimed, Unwanted

## Abstract

**Background:**

In Ethiopia, little is known about pregnancy among rural women. Proper maternal health care depends on clear understanding of the reproductive health situation. The objective of this study was to identify predictors of unintended pregnancy in rural eastern Ethiopia.

**Methodology:**

This study was part of pregnancy surveillance at Kersa Demographic Surveillance and Health Research Center, East Ethiopia. Pregnant women were assessed whether their current pregnancy was intended or not. Data were collected by lay interviewers using uniform questionnaire. Odds Ratio, with 95% confidence interval using multiple and multinomial logistic regression were calculated to detect level of significance.

**Results:**

Unintended pregnancy was reported by 27.9% (578/2072) of the study subjects. Out of which, 440 were mistimed and 138 were not wanted. Unintended pregnancy was associated with family wealth status (OR 1.47; 95% CI 1.14, 1.90), high parity (7 +) (OR 5.18; 95% CI 3.31, 8.12), and a longer estimated time to walk to the nearest health care facility (OR 2.24; 95% CI: 1.49, 3.39).

In the multinomial regression, women from poor family reported that their pregnancy was mistimed (OR 1.69; 95% CI 1.27, 2.25). The longer estimated time (80 + minutes) to walk to the nearest health care facility influenced the occurrence of mistimed pregnancy (OR 2.58; 95% CI: 1.65, 4.02). High parity (7+) showed a strong association to mistimed and unwanted pregnancies (OR 3.11; 95% CI 1.87, 5.12) and (OR 14.34; 95% CI 5.72, 35.98), respectively.

**Conclusions:**

The economy of the family, parity, and walking distance to the nearest health care institution are strong predictors of unintended pregnancy. In order to reduce the high rate of unintended pregnancy Efforts to reach rural women with family planning services should be strengthened.

## Introduction

An unintended pregnancy is mistimed pregnancy or unwanted one [1,2]. It is a public health problem which affects maternal and child health [3,4]. Maternal death, abortion, low birth weight baby, preterm birth and high infant mortality are attributed to unintended pregnancies [5].

Although several international declarations were passed on the problem, many women in sub-Saharan Africa are suffering from unwanted pregnancies [6-8]. Unsafe abortions accounted for 14% of all maternal deaths in sub-Saharan Africa, where half of the world's maternal deaths occur [9,10].

In Ethiopia, according to the DHS-2005, 16.2% and 18.7% of the study subjects reported that their last pregnancy was unwanted and mistimed, respectively [11].

According to the World Health Organization, the country has the fifth largest number of maternal deaths in the world [9]. The maternal mortality ratio (MMR) in Ethiopia was estimated to be 673 deaths per 100,000 live births in the year 2005 and unsafe abortions accounted for 32% of all the maternal deaths in in the country [9,11,12].

Although there are a number of contributing factors for the occurrence of unintended pregnancy, failure of the health care system to meet the demands for reproductive health services particularly that limit family size is recognized as the major one [13,14].

Unintended pregnancies is higher among women who were unmarried, lower economic status, at an early or late age of reproductive life, not using contraceptives consistently and attending formal education. In the Ethiopian DHS, many of the mistimed pregnancies occurred in the age group of less than 30 while the majority of unwanted pregnancies were in the age group of 30 and older [11,15-18].

Even though researches from other countries presented a detailed report on the issue, in Ethiopia, available literatures do not provide sufficient evidence and sub national level information are lacking; as a result statistics regarding the rural women is hardly available. Such evidence is an essential part in improving the reproductive health service delivery to the rural women.

## Methods

This study was part of pregnancy surveillance initiated in Kersa Demographic Surveillance and Health Research Center (KDS-HRC) in Kersa district, East Hararge Zone, Oromia region, Ethiopia [19]. It was conducted form December 2009 to November 2010. The study site has twelve Kebeles (the smallest administrative unit in Ethiopia) with 10,256 households and a population of 48,192. The crude death rate and birth rate in the study site were 11.9 and 29.1 per 1000 population, respectively.

The district has six health centers and all the Kebeles in the study site had one to two health extension workers who provide basic primary health care services, including family planning and antenatal health care. According to the district health office, the health care coverage of the district was 80% in 2010 [20].

The study was approved by the Federal Democratic Republic of Ethiopia, National Ethical Clearance Board having a reference number 3.10/313/03. The Study participants were informed about the study. An information sheet that depicts the nature of the study, objective, outcome, benefit and risks associated with the study was given to the study participants. A written informed consent was obtained from the participants of this study. A copy of the written consent is available for review by the Editor-in-Chief of this journal.

The aim of the study was to determine the level and predictors of unintended pregnancy. All pregnant women (2072) identified during the study period were selected as a study subjects. Data were collected using questionnaire adapted from DHS and other relevant research reports [11]. The participants were asked if they had intended to have the index pregnancy.

Current age, maternal education, residence, wealth status, ever used family planning; gravidity, parity, and estimated time needed to walk to the nearest health facility were the independent variables.

Pregnancy was defined as "intended" if the respondent said she wanted to be pregnant or "unintended" she did not want to be pregnant [1]. The pregnancy intention was recorded as "intended now" as 1, "intended not now" as 2, and "don't want pregnancy at all" as 3. Later, for regression analysis, "intended not now" and "don't want pregnancy" were grouped as "not intended" and recorded as "1" and those "intended now" were recorded as "0".

Furthermore, the unintended pregnancy was identified as either mistimed or unwanted.

Maternal education was grouped as "illiterate" for those who did not know how to read and write and all others as "literate" and was coded 0 and 1, respectively. The ten rural Kebeles were labeled as 'rural' and the two small towns (Kersa and Water) were labeled as 'rural towns' with a code of 0 and 1, respectively.

To determine the economic status of the women, a wealth index was generated using 33 variables. The wealth distribution was generated by applying principal components these variables [11,21]. The variables used included income, maternal occupation, ownership of durable assets, ownership of farm land, access to utilities and infrastructure such as sanitation, source of water, housing characteristics and the ownership of a bank saving/cooperative savings account [21,22]. Categorical variables were made dummy before initiating analysis. Finally, the wealth status was determined by using three groups, rich as 1 middle as 2, and poor as 3.

Gravidity, parity and the number of living children were regrouped as never (0), 1-4, 5-6 and 7 plus. Access to health care service was determined by inquiring the main source of health care services and the estimated time to walk to the nearest health care institution. The main sources of health care services were later grouped into three and coded as health extension/health post/clinic as 1, community health center as 2, and hospital and other health care facility as 3. The estimated time to walk to the nearest health facility was measured in minutes and grouped as less than 40, 40-79 and 80 plus minutes. This is to proxy five, ten and above ten kilo meter distance to the nearest health facility from the women's home.

The proportions of the independent variables were calculated against pregnancy intention and crude odds ratio was calculated. As current age, age of marriage, and age of first pregnancy tended to correlate, in the final analysis, only current age was entered. Again gravidity, parity, number of live children also tended to correlate and only parity was entered in the final analysis. Similarity, the main source of health care services and the estimated time to the nearest health care facility tended to correlate and the estimated time to the nearest health care facility was retained in the final model. Logistic regression was computed for variables with specific categories of socio-demographic/economic and reproductive health. Then, in the final model the variables selected for group analysis were put against pregnancy intention. To see the variation between mistimed and unwanted pregnancy, multinomial logistic regression was computed based on the wanted pregnancy.

## Results

The mean and the standard deviation of current age and age of first marriage were 24.5 (± 7) and 16.7(± 4.5), respectively. Of the study subjects 98.2% were rural dwellers and 85.5% were illiterate. 78% of the respondents reported the nearest Health Center was as the main source of their health care and on average they walked 45 minutes to reach the center (Table 1).

**Table 1:** Characteristics of study participants by pregnancy intention, Kersa, 2010

Characteristics		Total n = 2,072	Unintended n = 578	Intended n = 1,494	Crude OR	CI		p = value
Age	< 20 Years	159	21.4	78.6	1.000			
	20-29 Years	888	27.0	73.0	1.362	0.906	2.046	0.137
	30-39 Years	794	26.5	73.6	1.322	0.877	1.993	0.183
	40 + Years	231	40.7	59.3	2.523	1.591	4.000	0.000
Educational level	Literate	299	23.8	76.2	1.000			
	Illiterate	1773	28.6	71.4	1.286	0.967	1.711	0.084
Residence	Rural town	152	14.5	85.5	1.000			
	Rural	1920	29.0	71.0	2.049	1.517	3.825	0.000
Wealth status	Rich	589	23.8	76.2	1.000			
	Middle	730	25.5	74.5	1.096	0.852	1.411	0.474
	Poor	753	33.5	66.5	1.613	1.266	2.056	0.000
Ever use of FP	No	1,566	27.4	72.6	1.000			
	Yes	506	29.5	70.5	1.106	0.887	1.38	0.371
Parity	Never give birth	297	13.8	86.2	1.000			
	1-4 birth	1150	26.5	73.5	2.254	1.581	3.213	0.000
	5-6 birth	362	31.8	68.2	2.907	1.954	4.324	0.000
	7 + birth	263	44.5	55.5	5.004	3.322	7.537	0.000
Gravidity	Never give birth	100	15.0	85.0	1.000			
	1-4 birth	1242	24.7	75.3	1.861	1.059	3.270	0.031
	5-6 birth	602	32.6	67.4	2.736	1.540	4.860	0.001
	7 + birth	128	46.9	53.1	5.000	2.612	9.573	0.000
Main source of health care	Health Post	433	37.9	62.1	1.000			
	Health Center	1616	25.3	74.7	0.554	0.443	0.693	0.000
	Hospital/other	23	26.1	73.9	0.579	0.224	1.498	0.260
Time to walk to the nearest HF	< 40 min	568	23.1	76.9	1.000			
	40-79 min	1365	28.3	71.7	1.315	1.047	1.652	0.018
	80 + min	139	43.9	56.1	2.609	1.770	3.844	0.000

Crude odds ratio: are calculated using logistic regression. Intended pregnancy is coded as = 0 and Unintended as = 1

P values are based on results from crude logistic regression.

The pregnancy was unintended by 27.9% (95% CI; 25.9, 29.8) of the study participants. Unintended pregnancy was further split into mistimed and unwanted pregnancies. Among the 578 unintended pregnancies, 440 (76.1%) reported their pregnancy was mistimed and the remaining 138 (23.9%) reported their pregnancy as unwanted.

The proportion of unintended pregnancy increased as age increased and got pick among the age group of 40 and above, the rural dwellers and the women from poor family. The Unadjusted OR was of 5.52 (1.59, 4.00) for age 40 and above, 2.05(1.52, 3.83) for rural dweller and 1.61(1.27, 2.06) poor family (Table 1).

As the number of gravidity and parity increased, the current pregnancy tended to be unintended. Unintended pregnancy increased as the estimated distance from home to the nearest health care facility increased. Those who said the estimated distance was more than 40 minutes had more unintended pregnancies with unadjusted OR of 2.61 (1.77, 3.84) than those who reported less than 40 minutes (Table 1).

Unintended pregnancy was also measured by adjusting for some variables. The first model put the socio- demographic and economic variables together. When the four variables (age, education level, residence and wealth status) were controlled, women more than 40 years had unintended pregnancies with the odds ratio of 2.195 (1.37, 3.52). Women from the rural area and poor family reported that their current pregnancy was unintended, with the OR of 2.11 (1.31, 3.42) and 1.39 (1.08, 1.78), respectively (Table 2).

**Table 2:** Determinants of unintended pregnancy, Kersa district, Eastern Ethiopia, 2010

Characteristics		Model -I			Model -II			Final Model		
		**OR**	**CI**		**OR**	**CI**		**OR**	**CI**	

Age	< 20 Years	1.000						1.000		
	20-29 Years	1.298	0.858	1.964				0.979	0.631	1.517
	30-39 Years	1.186	0.777	1.81				0.703	0.445	1.11
	40 + Years	2.195**	1.369	3.518				1.304	0.787	2.16
										
Educational level	Literate	1.000						1.000		
	Illiterate	1.098	0.811	1.487				0.899	0.658	1.229
										
Residence	Rural town	1.000						1.000		
	Rural	2.111**	1.305	3.415				1.633	0.966	2.762
										
Wealth status	Rich	1.000						1.000		
	Middle	1.011	0.781	1.310				1.052	0.807	1.371
	Poor	1.387*	1.079	1.782				1.470**	1.136	1.902
										
Ever use of FP	No				1.000			1.000		
	Yes				1.009	0.805	1.264	1.091	0.863	1.378
										
Parity	Never give birth				1.000			1.000		
	1-4 birth				2.251***	1.577	3.214	2.324***	1.59	3.396
	5-6 birth				2.902***	1.946	4.327	3.153***	2.045	4.86
	7 + birth				4.997***	3.313	7.538	5.182***	3.307	8.121
Time to walk to the nearest HF	< 40 min							1.000		
	40-79 min							1.128	0.871	1.462
	80 + min							2.245***	1.485	3.394

LR chi2		48.04			70.29			125.47		
Pseudo R2		0.0196			0.0287			0.0511		
Prob > chi2		0.0000			0.0000			0.0000		
Log likelihood		-1202.5			-1191.4			-1163.8		

* = p < 0.05; ** = p < 0.01; *** = p < 0.001

In the second model, ever use of family planning and parity were put together. Parity showed its association with unintended pregnancy. The strongest association was among women who reported that seven parity above, with the adjusted OR of 4.99 (3.31, 7.54) (Table 2)

In the final model, from the first, second model and one additional variable (estimated time to walk to the nearest health facility), were put together. Economic status, parity and walking time showed significant association. The adjusted OR for women from poor family was 1.47 (1.13, 1.90) and for estimated time to walk to the nearest health facility 80 minutes and above was 2.25 (1.49, 3.39). Similar to model two, the strongest association was found among women of 7 and above parity with adjusted OR of 5.18 (3.31, 8.12) (Table 2).

Multinomial logistic regression was applied to examine the relative effects of the characteristics kept in the final model against the mistimed and unwanted pregnancies. Pregnancies were more mistimed among the rural dwellers and the women from poor family, with an adjusted OR of 1.96 (1.05, 3.68) and 1.69 (1.27, 2.25), respectively. Similarly, parity showed association with mistimed pregnancy and the strongest association was found among seven and above parity, with adjusted OR of 3.11 (1.87, 5.12). As time to walk to the nearest health facility increased, pregnancy was mistimed and a significant association was detected for those who reported to walk 80 and above minutes, with adjusted OR of 2.58 (1.65, 4.02) (Table 3).

**Table 3:** Determinants of mistimed and unwanted pregnancies, Kersa district, Eastern Ethiopia, 2010

Characteristics		Mistimed			Unwanted		
		**AOR**	**CI**		**AOR**	**CI**	

Age	< 20 Years	1.000			1.000		
	20-29 Years	0.935	0.592	1.477	1.534	0.444	5.293
	30-39 Years	0.645	0.399	1.043	1.380	0.399	4.773
	40 + Years	0.943	0.545	1.633	4.028*	1.142	14.209
							
Educational level	Literate	1.000			1.000		
	Illiterate	0.806	0.579	1.122	1.461	0.699	3.053
							
Residence	Rural town	1.000			1.000		
	Rural	1.964*	1.049	3.679	0.973	0.409	2.315
Wealth status	Rich	1.000			1.000		
	Middle	1.18	0.878	1.587	0.761	0.47	1.233
	Poor	1.689***	1.267	2.251	0.962	0.608	1.522
Ever use of FP	No	1.000			1.000		
	Yes	1.187	0.919	1.532	0.864	0.556	1.341
Parity	Never give birth	1.000			1.000		
	1-4 birth	2.337***	1.555	3.511	1.961	0.792	4.858
	5-6 birth	2.548***	1.586	4.093	6.122***	2.408	15.566
	7 + birth	3.111***	1.869	5.177	14.342***	5.717	35.983
Time to walk to the nearest HF	< 40 min	1.000			1.000		
	40-79 min	1.261	0.945	1.684	0.774	0.487	1.231
	80 + min	2.576***	1.649	4.023	1.436	0.685	3.010

LR chi2	225.47						
Pseudo R2	0.0730						
Prob > chi2	0.0000						
Log likelihood	-1431.51						

* = p < 0.05; ** = p < 0.01; *** = p < 0.001

Age, 40 and above, and parity showed association with unwanted pregnancies. Pregnancy was unwanted 4.028 (1.14, 14.21) times among women aged 40 and above than their counter parts. Pregnancy was unwanted among the higher parity women with the OR of 6.12 (2.4, 15, 57) for 5-6 parity and 14.342 (5.72, 35.98) for 7 plus (Table 3)

## Discussion

In this study unintended pregnancy was 27.9% among the study participants. It was associated with wealth, parity and distance to the nearest health facility. Women from poor family, those who reported estimated time to walk to the nearest health facility to be 80 minutes and above reported that their pregnancy was unintended. As parity increased, pregnancy became more and more unintended.

This study is a part of a follow-up of pregnant women in eastern Ethiopia [19]. It started by identifying whether a women was pregnant or not and then asked whether the current pregnancy was intended or not. Hence, there was a high probability of getting accurate responses compared to studies that depended on retrospective questioning, which were highly affected by memory lapse and the outcome of the pregnancy [23]. However, as reported previously, once pregnancy occurred there was a tendency to report that the pregnancy was intended [2,16].

The level of unintended pregnancy found in this study is lower than that of the Ethiopian DHS estimation [11]. This may be explained by the fact that the study was conducted in Muslim dominated area, where every child is taken as gift of God and the belief that every child is living his own fate is held. It is very important to get provincial level information in a big country such as Ethiopia that has very diverse traditional, cultural and religious denominations in order to effectively define the health needs of women.

Several socio-demographic and reproductive factors related to pregnancy intention were not studied because of the homogeneity of the study population. That is the majority were Muslim, all married, and from one ethnic group [15,16]. The Homogeneity actually self-controlled for the most potential confounders and helped to pin point the most important predictors of unintended pregnancy.

Even though age failed to show significant association with unintended pregnancy, in the multinomial logistic regression, age above 40 showed statistically significant association with unwanted pregnancies. This finding is in agreement with the Ethiopian DHS 2005 [11]. This is may be explained by the fact that in most instances women at this age have sufficient number of children and they were fatigued due to repeated pregnancies occurred at younger age [1].

Consistent with studies conducted in Nigeria and other places, women from the poor family showed a positive association with unintended pregnancy [17]. This may be explained by the fact that, though in theory health services are at the door steps of everyone [24-26], in reality they are not. This requires the women to travel longer that compromises family responsibility and costs a lot. As a result they are unable to get the drugs to control for their fertility.

Parity has maintained its association with unintended pregnancy both at the bivariate and multivariate analyses. In the multinomial logistic regression, women who had 5-6 parity and 7 or more reported that their pregnancy was unwanted with an adjusted OR of 6.12 (2.41, 15.57) and 14.34(5.72, 35.98), respectively (Table 3). In the rural setting as indicated in many writings [27,28], even though there is a strong belief saying *"to have more children is to mean to have a good amount of wealth." *it is clear that for women who already had four parities, the current pregnancy was out of their intention. It indicates that the toll of high unmet need for family planning is evident [29].

As the estimated time to walk to the nearest health facility increased, women reported that their pregnancy was unintended. In Ethiopia, Kebeles are staffed with health extension workers. They are expected to deliver 16 packages including family planning [24-26].

These health workers overburdened with different duties,[25,30] and family planning drugs are short in most instances. As a result, women who demand fertility control services are obliged to go further to the towns where the health centers and hospital are located. This is a very difficult mission for rural women due to family responsibility and cost of travel. However, literate and women who had better autonomy tended to demand maternal health service even if the distance was far [31].

## Conclusions and recommendations

The significant predictors of unintended pregnancy were high parity, long walking distance to nearest health facility and poor wealth status. The burden of unintended pregnancy was heavier for women who were already disadvantaged. Thus improving maternal health and reaching the maternal MDG targets by reducing unintended pregnancy requires coordinated efforts for improving access to health services in general and that of family planning in particular.

## Authors' information

**NA: **is a Reproductive Health professional working in Haramaya University. He is actively working on Kersa Demographic Surveillance and Health Research Center, Haramaya University, Ethiopia.

**YB: **is a senior professor of epidemiology and public health at Addis Continental Institute of Public Health. He has been teaching several courses in public health, epidemiology and research methods in various universities for more than two decades. He has collaborated with national and international colleagues in many research projects and coauthored over 100 publications. He has advised many doctoral students in Ethiopia and internationally.

**AW**: is Associate Professor at the Department of Epidemiology and Biostatistics of Addis Ababa University, Ethiopia. He has been teaching Biostatistics and Research methods for many years. He has worked as co-investigator and technical coordinator, for many trial studies and other researches. He has more than 40 publications on pear reviewed international and national journals and chapter author of different public health study reports.

## Competing interests

The authors declare that they have no competing interests.

## Authors' contributions

**NA, YB, and AW: **participated in all steps of the study from its inception to write up. They have reviewed and approved the submission of the manuscript
